# Identification and Validation of an 6-Metabolism-Related Gene Signature and Its Correlation With Immune Checkpoint in Hepatocellular Carcinoma

**DOI:** 10.3389/fonc.2021.783934

**Published:** 2021-11-15

**Authors:** He Ren, Wanjing Li, Xin Liu, Shuliang Li, Hao Guo, Wei Wang, Na Zhao

**Affiliations:** ^1^ Department of General Surgery, Tianjin Medical University General Hospital, Tianjin Medical University, Tianjin, China; ^2^ Department of Gastrointestinal Surgery, The Second People’s Hospital of Liaocheng, Linqing, China; ^3^ Department of Gastrointestinal Surgery, The Second Hospital of Liaocheng Affiliated to Shandong First Medical University, Linqing, China

**Keywords:** hepatocellular carcinoma, metabolism‐related genes, prognosis, immune checkpoint, tumor infiltrating

## Abstract

Hepatocellular carcinoma (HCC) is a common malignant tumor with relatively high malignancy and rapid disease progression. Metabolism-related genes (MRGs) are involved in the pathogenesis of HCC. This study explored potential key MRGs and their effect on T-cell immune function in the tumor immune microenvironment to provide new insight for the treatment of HCC. Of 456 differentially expressed MRGs identified from TCGA database, 21 were screened by MCODE and cytoHubba algorithms. From the key module, GAD1, SPP1, WFS1, GOT2, EHHADH, and APOA1 were selected for validation. The six MRGs were closely correlated with survival outcomes and clinicopathological characteristics in HCC. Receiver operating characteristics analysis and Kaplan-Meier plots showed that these genes had good prognostic value for HCC. Gene set enrichment analysis of the six MRGs indicated that they were associated with HCC development. TIMER and GEPIA databases revealed that WFS1 was significantly positively correlated and EHHADH was negatively correlated with tumor immune cell infiltration and immune checkpoint expression. Finally, quantificational real-time polymerase chain reaction (qRT-PCR) confirmed the expression of WFS1 and EHHADH mRNA in our own patients’ cohort samples and four HCC cell lines. Collectively, the present study identified six potential MRG biomarkers associated with the prognosis and tumor immune infiltration of HCC, thus providing new insight into the pathogenesis and treatment of HCC.

## Introduction

Hepatocellular carcinoma (HCC) is one of the most common aggressive malignancies worldwide and the third leading cause of cancer-related death (1). In 2021, estimated approximately 42,230 new cases were diagnosed and 30,230 deaths occurred from HCC in the United States (2). In China, HCC is the most commonly diagnosed cancer and the leading cause of cancer death in men <60 years of age, with 466,100 new cases and 422,100 deaths in 2015 (3). Currently, the treatment of HCC consists mainly of hepatectomy, transplantation, or ablation. Despite advances in the clinical treatment and diagnosis of HCC, recurrence rates remain high and survival rates remain poor (1). Therefore, innovative approaches to HCC treatment are urgently needed to reduce disease recurrence and death.

Advances in genomic and proteomic technologies have improved our understanding of the pathogenesis of malignant tumors. Metabolism refers to the orderly chemical reactions that occur in an organism to sustain life. Dysregulation of cellular metabolism is considered as an important factor associated with carcinogenesis (4). During tumorigenesis, cancer cells produce lactate *via* glycolysis (Warburg effect), which has a profound effect on the tumor microenvironment (TME) and the proliferation of cancer cells (5). Recent studies showed that alterations in metabolic pathways, such as lipid metabolism, were strongly associated with the development and progression of HCC, and metabolic processes played an important role in the occurrence and progression of HCC (6). The identification of metabolism-related genes (MRGs) and the development of bioinformatics technologies may lead to the identification of reliable biomarkers for the early diagnosis of HCC, as well as for predicting the progression and prognosis of this disease. In this study, we constructed a six-MRG prognostic signature based on The Cancer Genome Atlas (TCGA) database. We experimentally validated the mRNA expression levels of WFS1 and EHHADH in our own patients’ cohort and HCC cell lines, and further explored their expression patterns, potential biological functions, and immune infiltration levels in HCC. Recent studies showed that immune checkpoint inhibitor (ICI) therapies, such as those targeting programmed cell death 1 (PDCD1), cytotoxic T lymphocyte-associated protein 4 (CTLA4), and lymphocyte-activation gene 3 (LAG3) showed significant benefits in terms of survival compared with conventional therapies (7, 8). Identification of potential prognostic markers associated with therapeutic benefit may allow the individualization of immunotherapy for patients with HCC. Our findings suggested that *WFS1* and *EHHADH* played essential roles in tumor growth by triggering immune checkpoints in HCC, and are thus promising prognostic biomarkers for patients receiving immunotherapy.

## Materials and Methods

### Data Collection and Pre-Processing

HCC expression profiles included 373 HCC tissue samples and 50 peri-tumor samples and corresponding clinical information were obtained from TCGA (https://cancergenome.nih.gov/) and used as an analysis dataset. HCC data from International Cancer Genome Consortium (ICGC) included 212 HCC tissue samples and 177 peri-tumor samples) and four raw HCC-associated gene expression datasets, including GSE14250 (247 HCC tissue samples and 239 peri-tumor samples), GSE36376 (240 HCC tissue samples and 193 peri-tumor samples), GSE54236 (81 HCC tissue samples and 80 peri-tumor samples), and GSE107170 (146 HCC tissue samples and 104 peri-tumor samples), which were downloaded from NCBI-GEO database (https://www.ncbi.nlm.nih.gov/gds/), were used as an external validation dataset. The MRGs were acquired from GeneCards (https://www.genecards.org/), which provides comprehensive information on all annotated and predicted human genes. The term “metabolism” was used as a keyword for the search, and genes with relevance scores >5 were considered as MRGs.

### Differentially Expressed MRG Screening and Functional Analysis

Differentially expressed genes (DEGs) in TCGA cohort were screened using the edge R package. Threshold values of log_2_FC>1 and adjusted P <0.05 were considered statistically significant. Intersecting DEGs and MRGs were selected as key genes for HCC metabolism for further analysis. To investigate the biofunctional characteristics and potential pathways of the identified MRGs, Gene Ontology (GO) enrichment and Kyoto Encyclopedia of Genes and Genomes (KEGG) pathway analysis were performed by an online annotation, visualization, and integrated discovery database. (DAVID; https://david.ncifcrf.gov/) (9). P <0.05 was deemed statistically significant.

### Protein-Protein Interaction (PPI) Network Construction and Hub MRG Identification

The PPI network was constructed based on selected MRGs by the Search Tool for the Retrieval of Interacting Genes/Proteins (STRING) database and those with co-expression coefficients >0.4 were extracted (9). Thereafter, Cytoscape software was used to visualize and analyze molecular interaction networks (10). The Molecular Complex Detection plugin (MCODE) was then used to screen significant gene clusters, and a score >5 was used as the selection criterion. Hub genes were selected from the intersection of the top 120 genes and calculated with 10 topological analysis methods using the Cytoscape plugin cytoHubba. Finally, the results of the two algorithms were intersected to obtain the final hub MRGs.

### UALCAN Database and Kaplan-Meier Analysis

UALCAN is a comprehensive, user-friendly, and interactive web resource based on the TCGA dataset for analyzing cancer OMICS data (11). This database was used to analyze the differential expression of 21 MRGs in tumor/peri-tumor samples, and key genes were selected from the 21 MRGs that were correlated with multiple clinical disease characteristics. The Kaplan-Meier plotter could assess the effect of 54 k genes (mRNA, miRNA, protein) on survival in 21 cancer types including breast (n = 7,830), ovarian (n = 2,190), lung (n = 3,452), and gastric (n = 1,440) cancer (12). Sources for the database include TCGA etc. The prognostic value of the mRNA expression of 19 MRGs in HCC was assessed using the Kaplan-Meier plotter. A log P-value <0.05 was considered statistically significant.

### The cBioPortal for Cancer Genomics Analysis

The cBioPortal for Cancer Genomics (http://www.cbioportal.org/) is a freely accessible platform for interactive exploration of multi-cancer genomic datasets such as the TCGA database (13). The frequency of genetic alterations of six hub MRGs in patients with HCC and their association with survival outcomes were explored using cBioPortal.

### Construction of a Prognostic MRG Signature and Gene Set Enrichment Analysis (GSEA)

To evaluate the prognostic value of the six selected MRGs, we used the HCC clinical dataset from the TCGA database and implemented the six-gene signature *via* multivariate Cox regression analysis using the Sangerbox (http://sangerbox.com/Tool) tool. The integrated prognostic ROC, KM curve plotting tool for the relationship between risk score and gene expression developed by ggplot2 package based on R language on Sangerbox website performed multivariate Cox regression analysis on the six metabolism-related gene prognostic risk models. Risk classification thresholds were generally defaulted to the median. GSEA is a computational method for determining whether a set of *a priori* defined genes shows statistically significant consistent differences between two biological states. The HCC gene expression matrix from the TCGA database was downloaded and subjected to GSEA to predict potential hallmarks. After executing the permutation test with 1,000 permutations, gene sets with a P-value < 0.05 were considered statistically significant.

### Immune Infiltration Analysis

Tumor Immune Estimation Resource (TIMER; https://cistrome.shinyapps.io/timer/) is a comprehensive resource for the systematic analysis of immune infiltrates across diverse cancer types (14). In this study, the expression of six MRGs involved in tumor purity and immune infiltration abundance (B cells, CD4+ T cells, CD8+ T cells, neutrophils, macrophages, and DCs) in HCC was explored. The connection between immune infiltration and gene copy number variation (CNV) was also analyzed. The immune scores, stromal scores and tumor purity were calculated based on the ESTIMATE algorithm (15). The immunophenscore (IPS) was calculated on a 0-10 scale based on the expression of the representative genes or gene sets of the immunophenogram. Higher IPS was associated with stronger immunogenicity, indicating a better response to ICI. The IPSs of HCC patients were obtained from The Cancer Immunome Atlas (https://tcia.at/home).

### Relationship Between Hub MRGs and Immune Checkpoints of HCC

GEPIA (http://gepia.cancer-pku.cn/) is a web tool that provides customizable functions such as tumor/normal differential expression analysis, profiling according to cancer type or pathological stage, patient survival analysis, similar gene detection, correlation analysis, and dimensionality reduction analysis (16). The correlation of screened MRGs with immune checkpoints in HCC was assessed using the GEPIA database. Statistical significance was determined using R >0.1 and P <0.05 as filter criteria.

### Clinical Patient Samples Collection and Gene Expression Validation

A total of 10 patients with HCC diagnosed in the General Surgery Department of Tianjin Medical University General Hospital from October 2015 to July 2017 were collected. Tumor tissues and paired non-tumor control tissues have a distance > 5 cm. This study was approved by the ethics committee of Tianjin Medical University General Hospital. All subjects gave informed consent prior to participation in the investigation. Furthermore, four HCC cell lines were selected to further validate the expression levels of WFS1 and EHHADH. Total RNA was extracted using the TRIzol reagent (Invitrogen, CA, USA), and the concentration was measured using a spectrophotometer (NANODROP; Thermo Fisher Scientific, USA). Then, total RNA was reverse-transcribed to synthesize cDNA, which was used to perform qRT-PCR for two MRGs. β-actin was used as internal control, and fold change (2−^△△CT^) was used to express the relative gene expression levels. The primer sequences are listed in **Table 1**.

**Table 1:** mRNA PCR primer.

Gene name		Sequence of primer
WFS1		F: CCCTCAAGGTGTTCCAGGAC R: ACAGAGAGCAGGAAATGGGC
EHHADH		F:GTCAACGCGATCAGTACGAC R: CCTAGGAGCACTGAAGCCAC

### Statistical Analysis

Statistical analysis was performed using GraphPad Prism (version 8.0; San Diego, CA, USA). The Student’s *t*-test was used to compare differences between the tumor and peri-tumor groups. The data were considered statistically significant at P <0.05.

## Results

### Screening of Differentially Expressed MRGs and Enrichment Analysis

**Figure 1:**
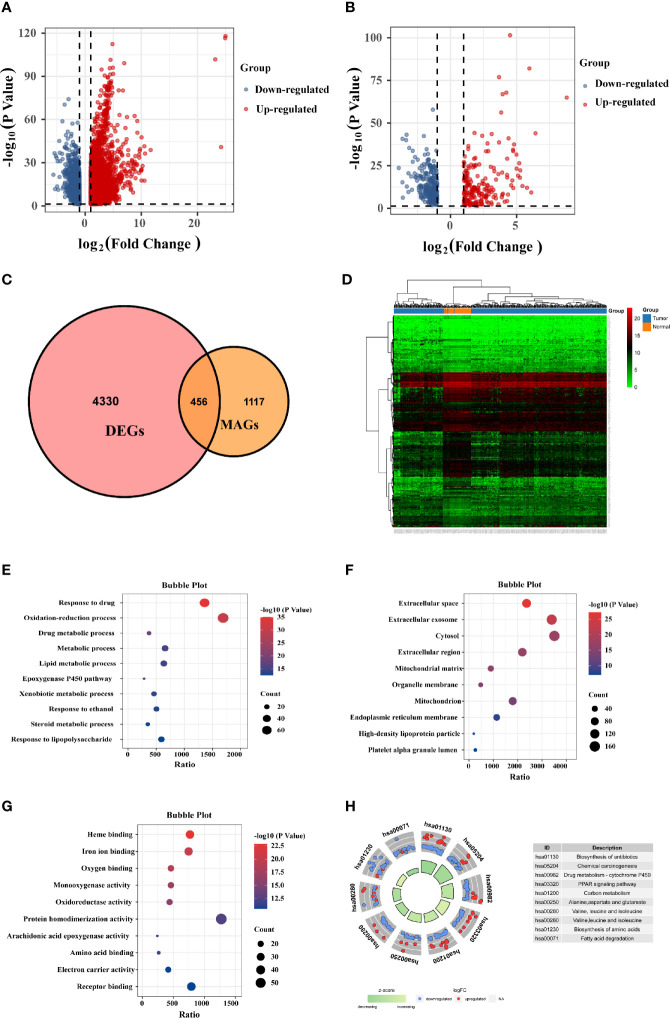
Identification of differentially expressed (DE)-MRGs and function enrichment analysis. **(A)** Volcano plot of DEGs in TCGA HCC samples. Genes with FDR <0.05 and FC >1.0 or <-1.0 are considered DEGs. Blue: downregulated genes; Red: upregulated genes. **(B)** Volcano plot shows 456 DE-MRGs in TCGA HCC samples. **(C)** Venn diagram indicating 456 DE-MRGs in the HCC cohorts of TCGA database. **(D)** Heat map shows the expression of 456 DE-MRGs in tumor and peri-tumor tissues. **(E–G)** Pathway enrichment by GO functional analysis. **(H)** The KEGG circle shows the scatter map of the logFC of the specified gene. Higher Z-score values indicate higher expression of the enrichment pathway.

The mRNA expression matrix of TCGA HCC patients was used to identify DEGs. Comparison of HCC and adjacent tissues in TCGA identified 4,786 DEGs, as shown in the volcano plot in **Figure 1A**. Analysis of the GeneCards website identified 1,857 MRGs with correlation scores >5, of which 456 were differentially expressed MRGs (DE-MRGs) in the TCGA HCC cohort (**Figure 1C**). Among them, 198 genes were downregulated and 258 genes were upregulated (**Figure 1B**); the heat map shows the distribution of the 456 MRGs (**Figure 1D**). The function of these DE-MRGs was examined by GO and KEGG analyses. The top 10 GO terms and KEGG pathways are listed in **Figure 1E**. For biological processes (BPs), DE-MRGs were mainly enriched in oxidation-reduction processes, lipid metabolic processes, metabolic processes, and xenobiotic metabolic processes (**Figure 1E**). Cellular component (CC) analysis showed that DEGs were mostly enriched in the extracellular exosome, cytosol, and high-density lipoprotein particle (**Figure 1F**). Regarding molecular function (MF), genes were primarily enriched in heme binding, monooxygenase activity, and arachidonic acid epoxygenase activity (**Figure 1G**). KEGG pathway enrichment analysis showed that MRGs were significantly associated with material synthesis and material metabolism signaling pathways, such as biosynthesis of amino acids, drug metabolism - cytochrome P450, and carbon metabolism (**Figure 1H**).

### Hub MRGs Screened Through the PPI Network

**Figure 2:**
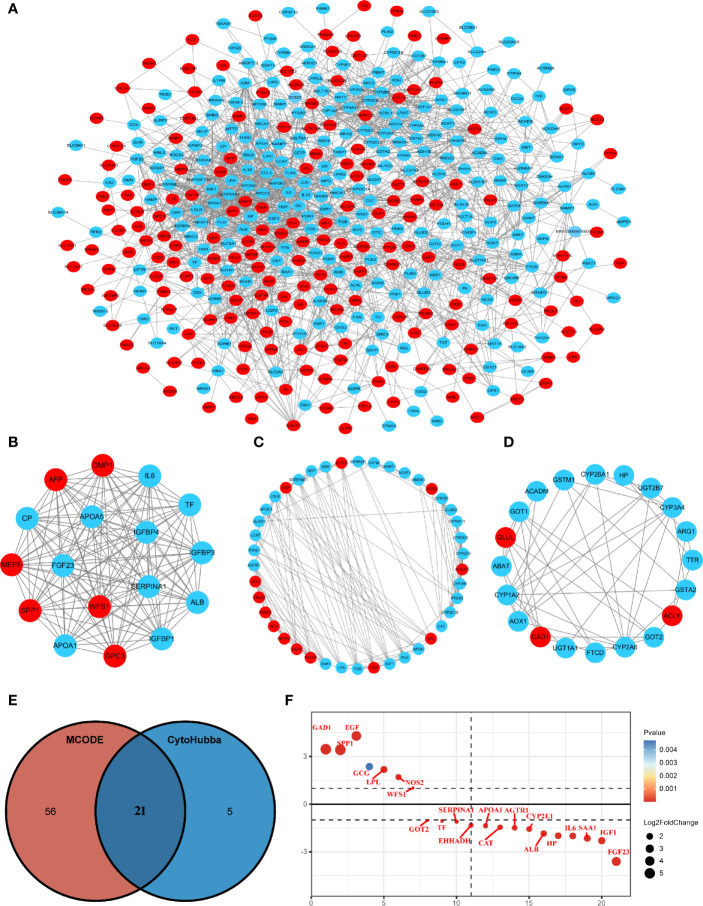
Protein-protein interaction (PPI) network construction and hub gene screening. **(A)** The interaction network between proteins coded by MRGs comprised 423 nodes and 2193 edges. Red circles represent upregulated genes and blue circles represent downregulated genes. **(B–D)** Three cluster modules were extracted by MCODE in PPI networks. **(E)** Venn diagram showing the hub genes obtained by intersecting the MCODE and cytoHubba algorithms. **(F)** Differential expression ranking of 21 hub MRGs.

The STRING online database and Cytoscape software were used to construct the PPI network, which contained 423 nodes and 2,193 edges (**Figure 2A**). Module analysis was performed using the MCODE plugin in Cytoscape software, and three modules were identified, comprising 77 hub genes (**Figures 2B–D**). Next, the cytoHubba plugin was used to identify the central gene, and 26 hub genes were selected by intersecting the results from the ten algorithms of cytoHubba. The results of the two algorithms were intersected to obtain the final 21 MRGs (**Figure 2E**), which are shown in **Figure 2F**.

### Gene Expression and the Prognostic Value of Identifying Hub MRGs in HCC

**Figure 3:**
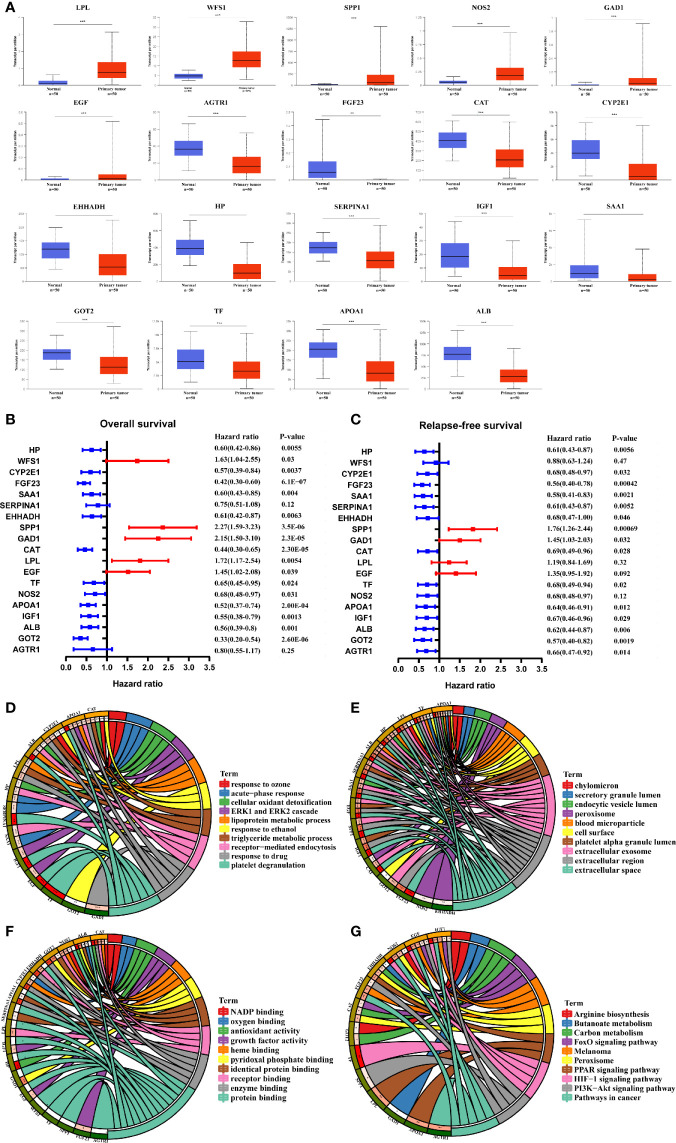
Validation of the expression and prognosis analysis of hub MRGs in HCC and their function enrichment. **(A)** UALCAN analysis of 21 DE-MRGs based on the TCGA database. ^*^P < 0.05; ^**^P < 0.01; ^***^P < 0.00001. **(B, C)** Forest plot of the univariate Cox regression analysis of OS and RFS with MRGs. P ≤ 0.05 was considered statistically significant. **(D)** Chord plot describing the relationship between 19 hub MRGs and GO terms of BP. **(E)** Chord plot depicting the relationship between 19 hub MRGs and GO terms of CC. **(F)** Chord plot showing the relationship between 19 hub MRGs and GO terms of MF. **(G)** Chord plot showing the relationship between 19 hub MRGs and KEGG pathways.

The transcriptional levels of the 21 screened MRGs were compared between HCC and peri-tumor tissues using UALCAN. The results showed statistically significant differences in the expression of 19 MRGs except GCG and IL6 (**Figure 3A**). Kaplan-Meier analysis showed that the 19 MRGs were related to clinical prognostic outcomes (**Figures 3B, C**). The 19 MRGs were subjected to repeat GO functional analysis and KEGG pathway analysis. BP term enrichment mainly involved triglyceride metabolic processes, lipoprotein metabolic processes, and cellular oxidant detoxification (**Figure 3D**). The CC terms included extracellular space, extracellular region, and extracellular exosome (**Figure 3E**). MF included antioxidant activity, pyridoxal phosphate binding, and receptor binding (**Figure 3F**). KEGG pathway analysis showed that the MRGs were associated with carbon metabolism, pathways in cancer, PPAR signaling pathway, and PI3K-Akt signaling pathway (**Figure 3G**). Most enriched pathways were involved in tumorigenesis and metabolic pathways.

### Validation of the Expression Levels of Six Hub MRGs

**Figure 4:**
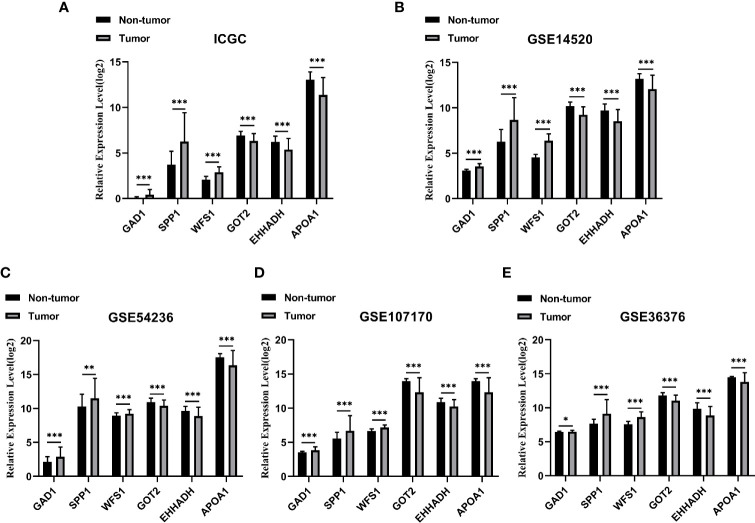
Verification of the six specifically expressed hub MRGs in five datasets. **(A)** Validation of six hub genes in the ICGC database. **(B)** Validation of six hub MRGs in GSE14520. **(C)** Validation of six hub MRGs in GSE54236. **(D)** Validation of six hub MRGs in GSE107170. **(E)** Validation of six hub MRGs in GSE36376. (^*^P < 0.05; ^**^P < 0.01; ^***^P < 0.001).

**Figure 5:**
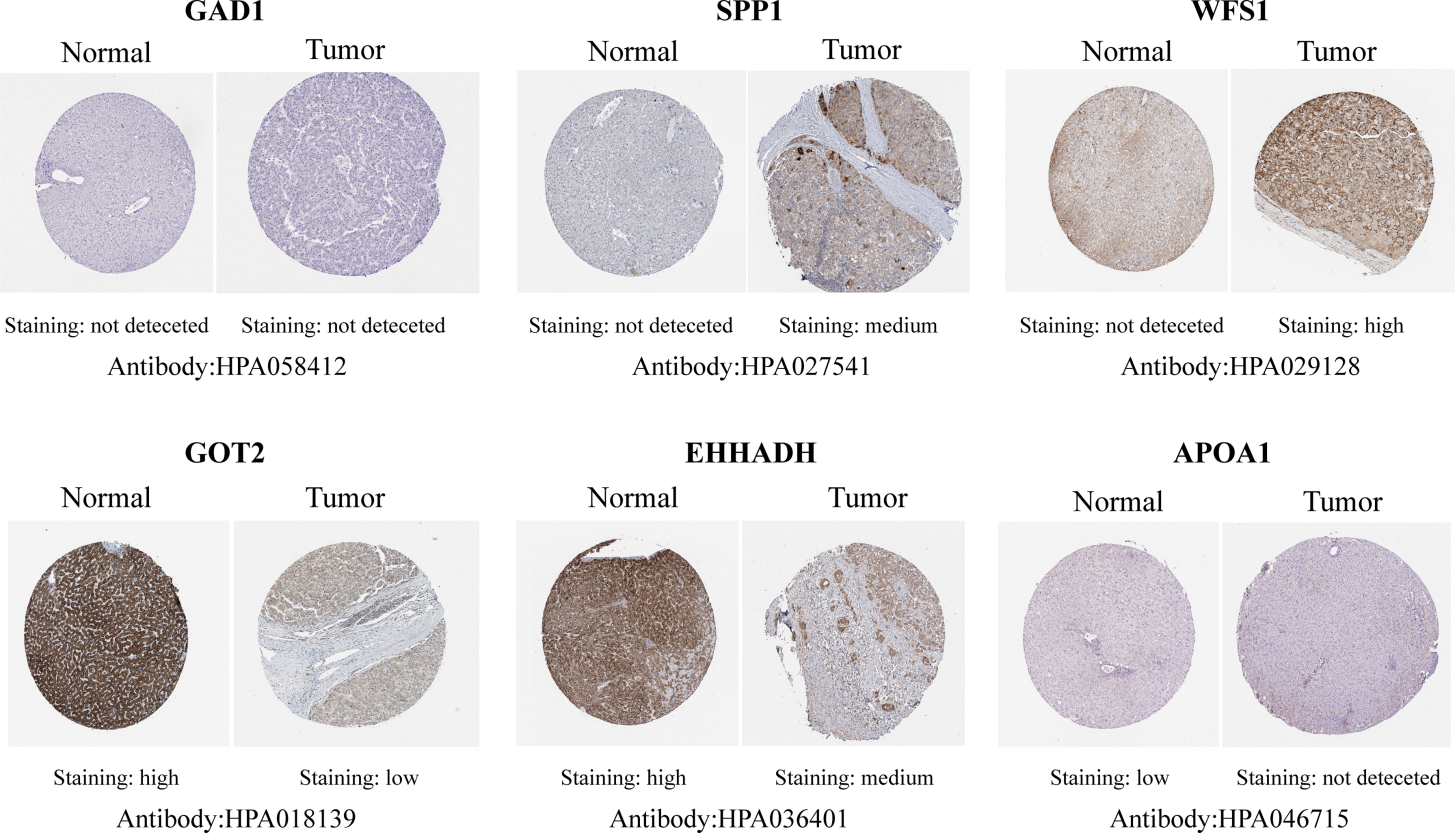
Representative immunohistochemistry staining validation of the expression of six MRGs. Protein expression levels of GAD1, SPP1, WFS1, GOT2, EHHADH, and APOA1 in HCC tissues were obtained from the Human Protein Atlas.

**Figure 6:**
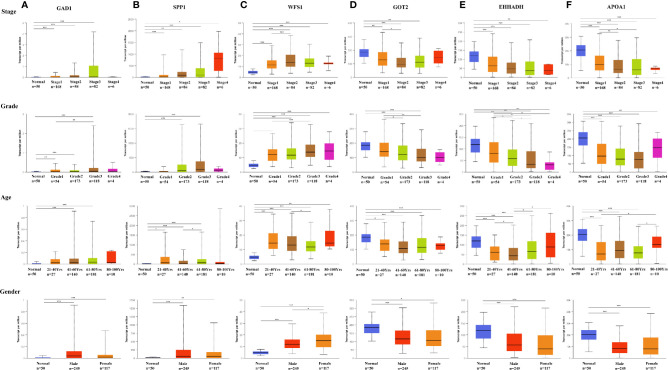
Expression of six MRGs in HCC subgroups stratified by clinical parameters in the UALCAN database. Boxplot showing the mRNA expression of six hub MRGs in HCC patients according to stage, grade, sex, and age. **(A)** GAD1, **(B)** SPP1, **(C)** WFS1, **(D)** GOT2, **(E)** EHHADH, **(F)** APOA1. ^*^P < 0.05; ^**^P < 0.01; ^***^P < 0.00001.

Among the 19 MRGs screened, we selected six (GAD1, SPP1, WFS1, GOT2, EHHADH, and APOA1) that have not been reported extensively in HCC, and their transcription levels and association with clinicopathological features were analyzed. GAD1, SPP1, and WFS1 were upregulated, whereas GOT2, EHHADH, and APOA1 were downregulated in HCC samples compared with peri-tumor controls, and this was consistent with the results in the ICGC, GSE14520, GSE54236, GSE107170, and GSE36376, validation datasets (**Figures 4A–E**). To verify the six MRGs at the translational level, protein expression data were obtained from the Human Protein Atlas (HPA) database. The protein expression levels of SPP1, WFS1, GOT2, EHHADH, and APOA1 showed a similar pattern to that of mRNA transcript levels (**Figure 5**). Furthermore, the six MRGs were significantly differentially expressed in HCC samples from patients with different tumor stage, grade, age, and sex according to the UALCAN analysis (**Figure 6**).

### Genetic Alterations of Six Screened MRGs in HCC

**Figure 7:**
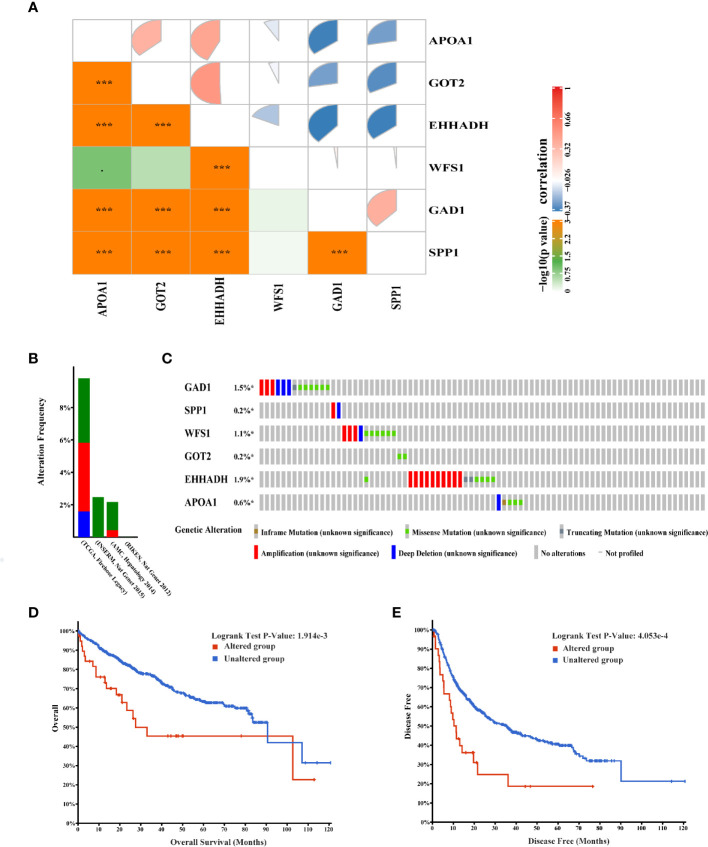
Genetic alterations associated with six hub MRGs in HCC. **(A)** Pearson’s correlation analysis of six MRGs in TCGA. *p < 0.05; ***p < 0.001. **(B)** Alterations of the six MRGs in four datasets: Liver Hepatocellular carcinoma (TCGA, Firehose Legacy), Hepatocellular carcinomas (INSERM, Nat Genet 2015), Liver Hepatocellular carcinoma (AMC, Hepatology 2014), Liver Hepatocellular carcinoma (RIKEN, Nat Genet 2012). **(C)** Alteration frequencies of six MRGs were based on the four datasets described above. **(D, E)** Kaplan-Meier plots comparing OS and DFS in cases with and without alterations in the six MRGs.

The correlation between the six hub MRGs was calculated using the TCGA-LIHC dataset, and Pearson’s correlation analysis indicated significant correlations among them (**Figure 7A**). Next, the genetic alterations of six MRGs in HCC patients were analyzed using the cBioPortal database in four datasets: Liver Hepatocellular carcinoma (TCGA, Firehose Legacy), Hepatocellular carcinomas (INSERM, Nat Genet 2015), Liver Hepatocellular carcinoma (AMC, Hepatology 2014), and Liver Hepatocellular carcinoma (RIKEN, Nat Genet 2012). The rate of genetic alterations of the six hub MRGs in the three datasets were 9.81% (37/377), 2.47% (6/243), and 2.16% (5/231), respectively (**Figure 7B**). The six MRGs had various genetic alterations, such as missense mutations, truncating mutations, amplifications, and deep deletions (**Figure 7C**). Cases with alterations in the six hub MRGs showed worse OS and DFS (P = 1.914E-3; P = 4.053E-4; **Figures 7D, E**).

### Evaluation and Validation of the Prognostic Signatures of Six MRGs

**Figure 8:**
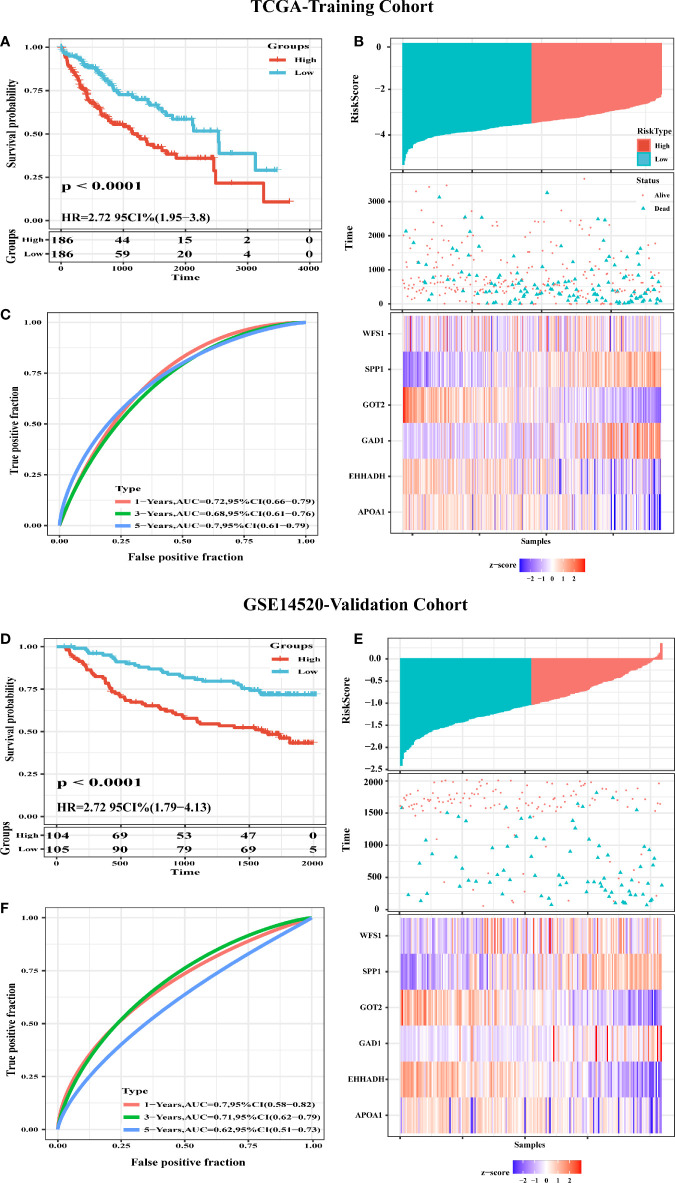
Construction and Validation of a six-MRG prognostic risk signature. **(A)** Survival curves for the low risk and high risk groups of the six-MRG signature in TCGA cohort. **(B)** ROC curve analysis of risk scores for predicting OS. **(C)** TCGA-LIHC dataset focused on risk score, survival time, survival status, and expression of the six-MRG signature. **(D)** Survival curves for the low risk and high risk groups of the six-MRG signature in GSE14520 cohort. **(E)** ROC curve analysis of risk scores for predicting OS. **(F)** GSE14520 dataset focused on risk score, survival time, survival status, and expression of the six-MRG signature.

To evaluate the classification performance of the six-MRG signature, we used the TCGA cohort as a training dataset. The risk scores for each sample were computed using the Sangerbox website, and the samples were divided into high and low risk groups according to the median risk score. Kaplan-Meier curves showed that patients in the high-risk group had markedly worse OS than those in the low-risk group (**Figure 8A**). The area under the curve (AUC) of the six gene signatures showed 1-, 3-, and 5-year OS rates of 0.72, 0.68, and 0.7, respectively, suggesting that the prognostic model had favorable sensitivity and specificity (**Figure 8C**). The risk score, survival time, survival status, and expression heatmap of the six-MRG signature are presented in **Figure 8B**. Moreover, the GSE14520 dataset were used for the validation of six metabolism-related gene signatures. Consistent with the results of the TCGA database, the Kaplan-Meier curve showed that patients in the high-risk group exhibited markedly worse OS than the low-risk group (**Figure 8D**). The AUC of the six gene signatures for 1-, 3- and 5-year OS rates were 0.7, 0.71 and 0.62 (**Figure 8F**). The risk score, survival time, survival status, and gene expression heatmap of six prognostic MRGs signature were shown in **Figure 8E**.

### Exploration of Significant Pathways for Six MRGs by GSEA

**Figure 9:**
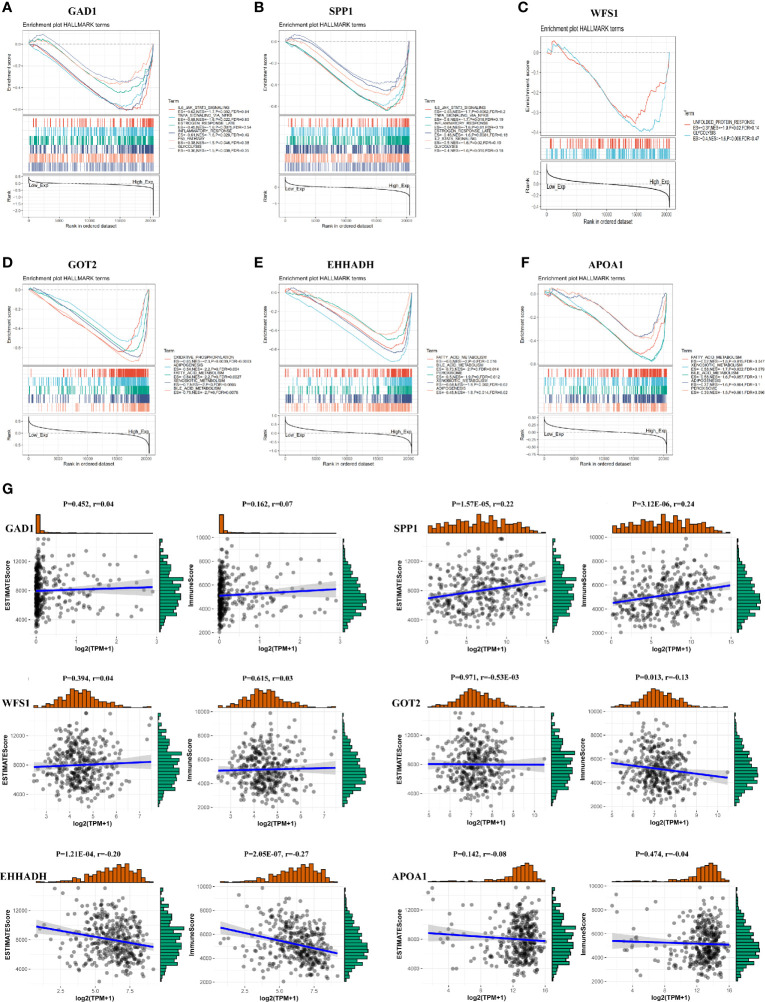
GSEA and immune/stromal/Estimate score for six MRGs in HCC. **(A–F)** Top six gene sets according to GSEA enrichment scores for GAD1, SPP1, WFS1, GOT2, EHHADH, and APOA1. **(G)** Association between immune/stromal/Estimate score and six MRGs after ESTIMATE algorithm processed.

To further investigate the potential functions of the six MRGs in HCC, we performed hallmark analysis by GSEA using the TCGA-LIHC dataset. GAD1 and SPP1 were enriched in the IL6-JAK-STAT3 signaling pathway, estrogen-response-late pathway, inflammatory-response pathway, TNF-α-signaling-via-NF-κB pathway, and glycolysis pathway (**Figures 9A, B**). WFS1 was enriched in the unfolded protein-response and glycolysis pathways (**Figure 9C**). GOT2, EHHADH, and APOA1 were all enriched for bile-acid-metabolism, fatty-acid-metabolism, xenobiotic-metabolism, peroxisome, and adipogenesis (**Figures 9D–F**). The pathways were markedly enriched in high risk samples and most of them were involved in metabolism and carcinogenesis.

### Association of the Six MRGs With Intratumor Immune Cell Infiltration

**Figure 10:**
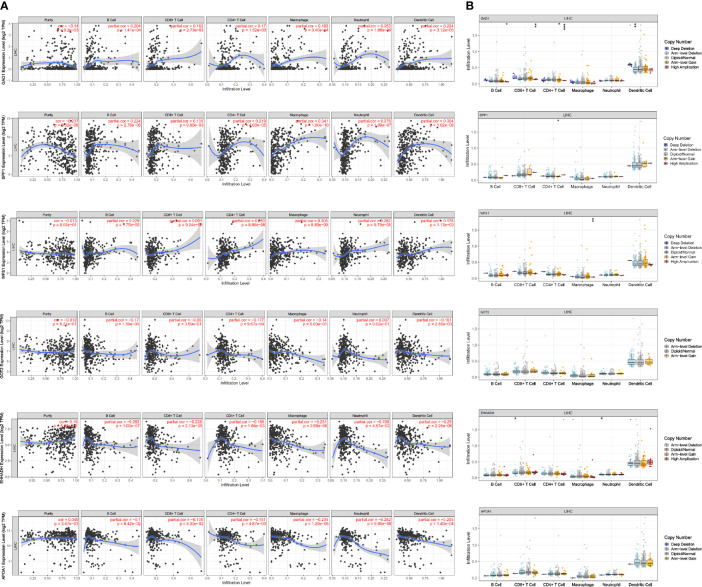
Association of mRNA expression of six MRGs with immune infiltration level in HCC. **(A)** Correlation of MRGs including GAD1, SPP1, WFS1, GOT2, EHHADH, and APOA1 with tumor purity and infiltration of B cells, CD8+ T cells, CD4+ T cells, macrophages, neutrophils, and dendritic cells. **(B)** The influence of copy number variation of GAD1, SPP1, WFS1, GOT2, EHHADH, and APOA1 on the distribution of diverse immune cells. *p < 0.05; **p < 0.01; ***p < 0.001.

To detect potential correlation between Estimate score/immune/stromal and six MRGs, the ESTIMATE algorithm was used to conduct the calculations. The results showed that SPP1, GOT2, and EHHADH were significantly associated with immune score or stromal score (**Figure 9G**).Then, the role of the six MRGs in the tumor immune microenvironment in HCC was examined by assessing the correlation between the expression of the six MRGs and infiltrated immune cell types and tumor purity using the TIMER database. The expression of GAD1 (r = -0.14, P = 9.2E^-03^) and SPP1 (r = -0.237, P = 8.32E^-06^) was negatively correlated, whereas EHHADH was positively correlated (r=-0.15, P =5.36E^-03^) with tumor purity (**Figure 10A**). GAD1, SPP1, and WFS1 were markedly positively correlated with the infiltration of six immune cell subtypes (B cells, CD8+ T cells, CD4+ T cells, macrophages, neutrophils, and DCs). The expression of the other three MRGs (GOT2, EHHADH, and APOA1) was significantly negatively correlated with the infiltration of six immune cell subtypes (**Figure 10A**). Also, the correlation and estimated statistical significance between six MRGs expression and other immune cell signature infiltration were displayed in **Supplementary Table 1**. The results showed that six MRGs were significantly correlated with the majority of immune cells such as NK cells, Tregs, and T cell follicular cell. The relationship between the CNV of six hub MRGs and tumor immune infiltrating cells is shown in **Figure 10B**.

### The Expression of WFS1 and EHHADH Is Correlated With Immune Checkpoints

Immune checkpoints are responsible for tumor immune escape. Considering the potential function of MRGs in HCC, we examined the relation of the six MRGs with the major immune checkpoints associated with HCC (PDCD1, CD276, CTLA-4, LAG3, TIM3, OX40, and TIGIT) (17, 18). The results demonstrated that WFS1 and EHHADH were significantly associated with immune checkpoint biomarkers. As shown in **Table 2**, relative WFS1 expression was significantly positively correlated with PDCD1, LAG3, TIM3, OX40, TIGIT, and CD276 levels. The tumor samples were then ranked according to the median level of WFS1 expression. The upper median was labeled WFS1-high and the lower median was labeled WFS1-low. The expression of PDCD1, LAG3, TIM3, OX40, TIGIT, and CD276 was significantly higher in the WFS1-high sample group than in the WFS1-low expression group (**Table 3**). EHHADH expression was significantly negatively correlated with T cell exhaustion markers in HCC, which showed a significant decrease in the EHHADH-high expression group (**Tables 2**, **3**). We then sought to determine whether the characteristics of the WFS1 and EHHADH genes could provide therapeutic value. We used IPS to predict the effectiveness of ICI in patients which were divided into high- and low-risk groups based on WFS1 and EHHADH gene expression levels. The low-risk group exhibited higher IPS (**Supplementary Figures 1A, B**). The WFS1 low-risk group exhibited higher IPS-PD1/PDL1/PDL2 scores than the high-risk group, representing higher immunogenicity and thus predicting a better response to ICI (**Supplementary Figure 1A**). Taken together, these results suggested that WFS1 and EHHADH played a critical role in tumor immune escape, which was involved in the carcinogenesis of HCC.

**Table 2:** Correlation of WFS1 and EHHADH expression with PDCD1, LAG3, TIM3, OX40, TIGIT, CD276, and CTLA4 expression in HCC.

NameCorrelation	PDCD1	LAG3	TIM3	OX40	TIGIT	CD276	CTLA4
**WFS1**	r=0.17 ***P*=0.00083**	r =0.11 ***P*=0.036**	r =0.36 ***P*=7.1E-13**	r =0.29 ***P*=9.5E-09**	r =0.11 ***P*=0.003**	r =0.41 ***P*=1.2E-16**	–
**EHHADH**	r =-0.38 ***P*=5.4E-14**	r =-0.25 ***P*=7.1E-07**	r =-0.25 ***P*=9.1E-07**	r =-0.4 ***P*=2.4E-15**	r=-0.23 ***P*=7.7E-06**	r =-0.32 ***P*=1.9E-10**	r=-0.38 ***P*=8.6E-14**

Bold values indicate that P < 0.05 was considered statistically significant.

**Table 3:** Comparison of the levels of PDCD1, LAG3, TIM3, OX40, TIGIT, CD276, and CTLA4 between the WFS1, EHHADH-high and –low subgroups.

parameter	WFS1-Low	WFS1-High	*t-value*	*p-value*	EHHADH-Low	EHHADH -High	*t*-value	*p-value*
	x̄ ± s				x̄ ± s			
**PDCD1**	0.7020 ± 0.2203		3.186	**0.0016**	-1.818 ± 0.2018		9.012	**<0.0001**
**LAG3**	0.3453 ± 0.1746		1.977	**0.0488**	-0.8867 ± 0.1701		5.212	**<0.0001**
**TIM3**	0.2819 ± 0.1373		2.053	**0.0408**	-0.9243 ± 0.1328		6.958	**<0.0001**
**OX40**	0.6189 ± 0.1208		5.125	**<0.0001**	-0.9750 ± 0.1148		8.495	**<0.0001**
**TIGIT**	0.3756 ± 0.1862		2.017	**0.0444**	-1.161 ± 0.1831		6.337	**<0.0001**
**CD276**	0.2218 ± 0.06859		3.234	**0.0013**	-0.5354 ± 0.06435		8.321	**<0.0001**
**CTLA4**	–		–	–	-1.649 ± 0.1818		9.071	**<0.0001**

Bold values indicate that P < 0.05 was considered statistically significant.

### Experimental Validation of WFS1 and EHHADH mRNA Expression

**Figure 11:**
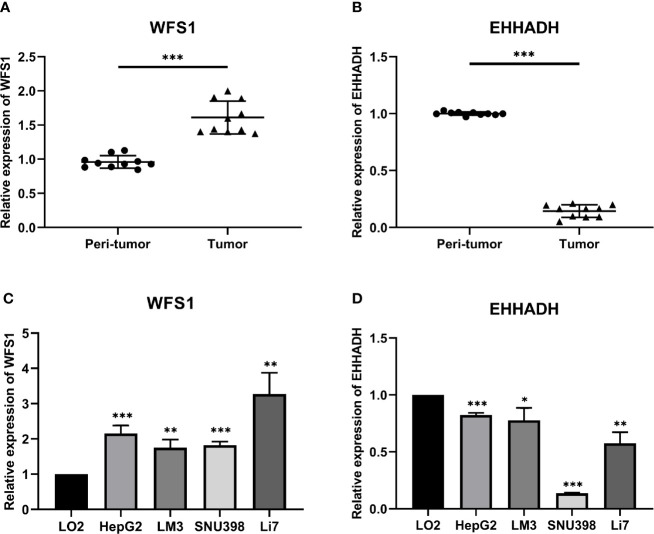
Relative expression levels of WFS1 and EHHADH detected by qRT-PCR. **(A)** Scatter plots of WFS1 were constructed using qRT-PCR data of our own patients’ cohort. **(B)** Scatter plots of EHHADH were constructed using qRT-PCR data of our own patients’ cohort. **(C)** The expression levels of WFS1 in LO2 and 4 HCC cell lines (HepG2, LM3, SNU-398, and Li7) detected by qRT-PCR. **(D)** The expression levels of EHHADH in LO2 and 4 HCC cell lines (HepG2, LM3, SNU-398, and Li7) detected by qRT-PCR. ^*^P < 0.05; ^**^P < 0.01; ^***^P < 0.001.

The results of bioinformatics analysis were confirmed by qRT-PCR in 10 paired HCC with peri-tumor samples and 4 HCC cell lines, the LO2 cell line was used as the control. The results showed that WFS1 was significantly upregulated (**Figures 11A, C**), whereas EHHADH (**Figures 11B, D**) was significantly downregulated in 10 HCC samples and 4 HCC cell lines, which was consistent with the bioinformatics results.

## Discussion

The age of incidence of HCC has decreased gradually in association with higher malignancy, rapid disease progression, and lack of obvious clinical symptoms in the early stages of the disease. Therefore, elucidating the molecular mechanisms of hepatocarcinogenesis may provide important clues for finding effective therapeutic targets or for identifying promising prognostic biomarkers. In recent years, there has been an increased interest in the study of metabolic alterations and dysregulation of the TME (5). Increasing evidence suggests that MRGs play a pivotal role in the development and progression of HCC (18). Considering the importance of the metabolic environment in tumor development, it is vital to identify metabolism-related HCC biomarkers.

In this study, we detected 456 DE-MRGs in TCGA dataset, including 198 downregulated and 258 upregulated MRGs. The 21 most significant hub MRGs were identified using the cytoHubba and MCODE algorithms with Cytoscape software. Of these, 19 MRGs were most significantly associated with transcriptional levels and clinical prognostic outcomes in HCC. Among these 19 hub MRGs, we selected six that have not been reported extensively in HCC, namely GAD1, SPP1, WFS1, GOT2, EHHADH, and APOA1, to investigate their diagnostic and prognostic value. GAD1 catalyzes the synthesis of γ-aminobutyric acid inhibitory neurotransmitters (19), and it is overexpressed in various neoplastic tissues, although its expression in HCC has not been reported. SPP1 is a secreted glycophosphoprotein that is associated with multiple biological functions, including cell adhesion, migration, and invasion (20, 21); however, its role in HCC remains unclear. The WFS1 gene, encoding wolframin (WFS1), causes endoplasmic reticulum stress and is linked to a rare autosomal-recessive disorder known as Wolfram syndrome (22). However, its role in the development of HCC remains unclear. Similarly, the prognostic role of GOT2, EHHADH, and APOA1 in HCC remain so far obscure. Compared with peri-tumor samples, GAD1, SPP1, and WFS1 were significantly upregulated in tumor tissues, and GOT2, EHHADH, and APOA1 were significantly downregulated in tumor tissues. The reliability of the expression of the six hub MRGs was further assessed in five datasets, including ICGC, GSE14520, GSE36376, GSE54236, and GSE107170, and the results were consistent with the cohort expression levels.

UALCAN analysis indicated that the transcription levels of GAD1, SPP1, and WFS1 were markedly higher in HCC tissues than in peri-tumor control tissues in the subgroup analyses. Increased gene expression was associated with worse survival, suggesting that these three MRGs function as oncogenes in HCC. In contrast, GOT2, EHHADH, and APOA1 were associated with better survival outcomes and were significantly downregulated in HCC tissues, as well as positively correlated with tumor stage, grade, age, and sex, suggesting that these three MRGs were tumor suppressor genes.

Furthermore the gene alteration frequencies of the above six MRGs in HCC were explored. Most of the six MRGs showed genetic alterations including gene amplifications, deep deletions, and missense mutations. Moreover, cBioPortal analysis showed that alterations in the six MRGs correlated with worse OS (p = 1.914E^-3^) and DFS (p = 4.053E^-4^). These results indicated that genetic alterations of the six MRGs may serve as valuable biomarkers for HCC diagnosis and treatment. In this study, we assessed the classification performance of the six-MRG signature model using TCGA database. The ROC curves showed that the six-MRG signature performed well in predicting the prognosis of HCC patients, especially for OS within 1, 3, and 5 years, implying that all six MRGs can be used as biomarkers to predict prognosis with good sensitivity and specificity. The prognostic value of this six MRG signatures was further successfully validated in the GSE14520 dataset. Moreover, the GSEA results suggest that many HALLMARK pathways related to tumorigenesis and metabolism, such as IL6-JAK-STAT3-signaling, TNF-α-signaling-via-NF-κB, glycolysis pathway, bile-acid-metabolism, and fatty-acid-metabolism were enriched in the expression groups of the six MRGs, suggesting their contribution to HCC progression through metabolic regulation. The IL6-JAK-STAT3 pathway consists of extracellular IL-6 ligands that activate the IL-6 receptor, which phosphorylates JAK, which in turn phosphorylates STAT3. Phosphorylated STAT3 dimerizes and translocates into the nucleus to induce the expression of genes with various tumor-promoting properties (23). Therefore, exploring biomarkers related to the IL6-JAK-STAT3 pathway may be potentially valuable for the detection and treatment of cancer. In addition, NF-κB is a family of inducible transcription factors that play multiple evolutionarily conserved roles in the immune system (24).TNF-α cytokines induce rapid transcription of genes regulating cell survival, proliferation and differentiation mainly through activation of the NF-κB pathway (25). Previous studies have reported that TNF-α activation of NF-κB is one of the most characteristic tumorigenic cytokines in hepatocellular carcinogenesis (26). Deep understanding of TNF-α-signaling-via-NF-κB pathway would throw lights on the interaction of MRGs and tumor progression. Similarly, glycolysis pathway, bile-acid-metabolism, and fatty-acid-metabolism also involved in tumor progression through affecting cellular metabolic processes (27–29).

Tumor-infiltrating immune cells affect the efficacy of radiotherapy, chemotherapy, or immunotherapy, and thus the prognosis of cancer patients (30–32). We showed that GAD1, SPP1, and WFS1 were markedly positively correlated with diverse immune cells, including B cells, CD8+T cells, CD4+T cells, macrophages, neutrophils, and dendritic cells in HCC. In contrast, GOT2, EHHADH, and APOA1 were significantly negatively correlated with tumor infiltrating immune cells. These findings indicated that tumor immune infiltration may partially account for the oncogenic role of metabolism-related genes in HCC. Additionally, the efficacy of immunotherapy requires not only sufficient immune cell infiltration in the TME, but also depends on the adequate expression of immune checkpoints (33). Compared with conventional therapies, ICI therapies such as those targeting programmed death ligand 1 (PD-L1), PDCD1, and CTLA4, have shown significant survival benefits (17). The activity of the immune system is primarily regulated by immune cells called T cells. In the TME, the response of T cells is regulated by a group of cell surface molecules called immune checkpoints. Cancer cells can evade the immune system, mainly by overexpressing suppressive immune checkpoints that cause T-cell exhaustion, thereby evading T-cell attack (34). In the HCC tumor microenvironment, PDCD1 is expressed in CD8+ T cells, and when activated, PDCD1 inhibits T cell migration, proliferation and secretion of cytotoxic mediators, thereby blocking the “cancer immune cycle” (35). In human liver cancer tissues, PD-L1 expression was mainly expressed in Kupffer cells, and PD-L1+Kupffer cells interacted with PD-1+CD8+ T cells, resulting in hepatocellular carcinoma effector T cell dysfunction, suggesting that PD-L1/PD-1 immune checkpoints could be used as targets for the treatment of HCC (35, 36). LAG-3 and T-cell immunoglobulin and mucin structural domain molecule-3 (Tim-3), are also upregulated on specific CD8+ T cells in various cancer types and are also involved in the progression of hepatocellular carcinoma. Recently, LAG3 expression was previously found to be significantly higher in CD8+ tumor-infiltrating helper T cells and CD8+ cytotoxic T cells than in tumor-free liver tissue and blood from patients with hepatocellular carcinoma (37). T-cell immunoglobulin-3 (Tim-3) is a type I transmembrane protein that is expressed by most tumor-infiltrating Tregs in the tumor setting and appears to represent a specific tissue-resident Treg subpopulation with enhanced suppressive activity (38). It has also been reported that Tim-3 is strongly expressed on CD4+ and CD8+ T cells obtained from hepatocellular carcinoma lesions, and these data suggest that the functional relationship between Tim-3 and different T cells may affect the prognosis of HCC (37). TIGIT was identified as a co-receptor expressed mainly by activated and regulatory T cells (Treg) and NK cells (39). It has been found that TIGIT can contribute to the progression of hepatocellular carcinoma by modulating the immunosuppressive effect of cytokines produced by CD155 on CD8 T cells (40). Similarly, high expression of OX40, CD276, CTLA4 was significantly associated with poor prognosis of hepatocellular carcinoma (41–43).

Cancer cells are highly dependent on stress adaptation and altered metabolic states for tumor cell proliferation (44). Metabolic changes in tumor cells can also affect the composition and function of tumor-infiltrating lymphocytes residing in the same microenvironment, such as T-cell exhaustion, which in turn causes tumor progression (44). Many tumors are capable of evading the immune system, primarily by overexpressing suppressive ligands that inhibit T-cell attack. Consequently, fewer and impaired T cells are found in patients with HCC, which contributes to the progression of this cancer. We therefore assessed the relationship between the six MRGs and immune checkpoints. The results showed that high expression of WFS1 was closely associated with PDCD1, LAG3, TIM3, OX40, TIGIT, and CD276 in HCC, and low expression of EHHADH was negatively correlated with PDCD1, LAG3, TIM3, OX40, TIGIT, CD276, and CTLA-4. This indicated that WFS1 or EHHADH could promote inhibitory receptor induction and exhaustion and that targeting both may increase the efficacy of immunotherapy for HCC.

## Conclusion

This study identified six MRGs that were closely related to the tumorigenesis and prognosis in HCC by multi-cohort dataset and comprehensive bioinformatics analyses. The current findings suggested that WFS1 and EHHADH exerted their oncogenic effects by activating immune checkpoints, indicating that their expression levels may serve as biomarkers for predicting the efficacy of ICI in HCC patients, which should be further explored in the future. The elucidation of the underlying mechanism and the validation of our observations in clinical trials will help develop more effective treatments for HCC patients.

## Data Availability Statement

The original contributions presented in the study are included in the article/**Supplementary Material**. The analyzed data in this study can be found here: GEO (http://www.ncbi.nlm.nih.gov/geo/) and TCGA (https://portal.gdc.cancer.gov/). Further inquiries can be directed to the corresponding author.

## Author Contributions

HR and NZ performed bioinformatics analysis and paper draft. HG, WW, and WL revised the images. XL and SL performed the literature search and data analysis. NZ revised the manuscript. All authors contributed to the article and approved the submitted version.

## Funding

This work was supported by the Tianjin Health Commission Science-technology Projects (grant number ZC20186).

## Conflict of Interest

The authors declare that the research was conducted in the absence of any commercial or financial relationships that could be construed as a potential conflict of interest.

## Publisher’s Note

All claims expressed in this article are solely those of the authors and do not necessarily represent those of their affiliated organizations, or those of the publisher, the editors and the reviewers. Any product that may be evaluated in this article, or claim that may be made by its manufacturer, is not guaranteed or endorsed by the publisher.

## Supplementary Material

Supplementary Figure 1 Comparison of WFS1 and EHHADH response to immunophenoscore treatment. **(A)** IPS, IPS- CTLA4, IPS- PD1/PDL1/PDL2, and IPS- PD1 PD1/PDL1/PDL2+CTLA4 in WFS1 signature high- risk group and low- risk group. **(B)** IPS, IPS- CTLA4, IPS- PD1/PDL1/PDL2, and IPS- PD1 PD1/PDL1/PDL2+CTLA4 in EHHADH signature high- risk group and low- risk group.

## Abbreviations

MRGs, metabolism-related genes; HCC, Hepatocellular carcinoma; ICGC, International Cancer Genome Consortium; GEO, Gene Expression Omnibus; TCGA, The Cancer Genome Atlas; PPI, Protein–protein interaction; DEGs, Differentially expressed genes; GO, Gene Ontology; KEGG, Kyoto Encyclopedia of Genes and Genomes; TME, The Tumor Microenvironment; GSEA, Gene Set Enrichment Analysis; OS, overall survival; RFS, relapse-free survival; DFS, disease-free survival; HPA, Human Protein Atlas.
