# Supplementary figures and images for: Identification and Validation of an 6-Metabolism-Related Gene Signature and Its Correlation With Immune Checkpoint in Hepatocellular Carcinoma
Source: Front Oncol. 2021 Nov 15;11:783934. doi: 10.3389/fonc.2021.783934 (PMC8634254; doi:10.3389/fonc.2021.783934)

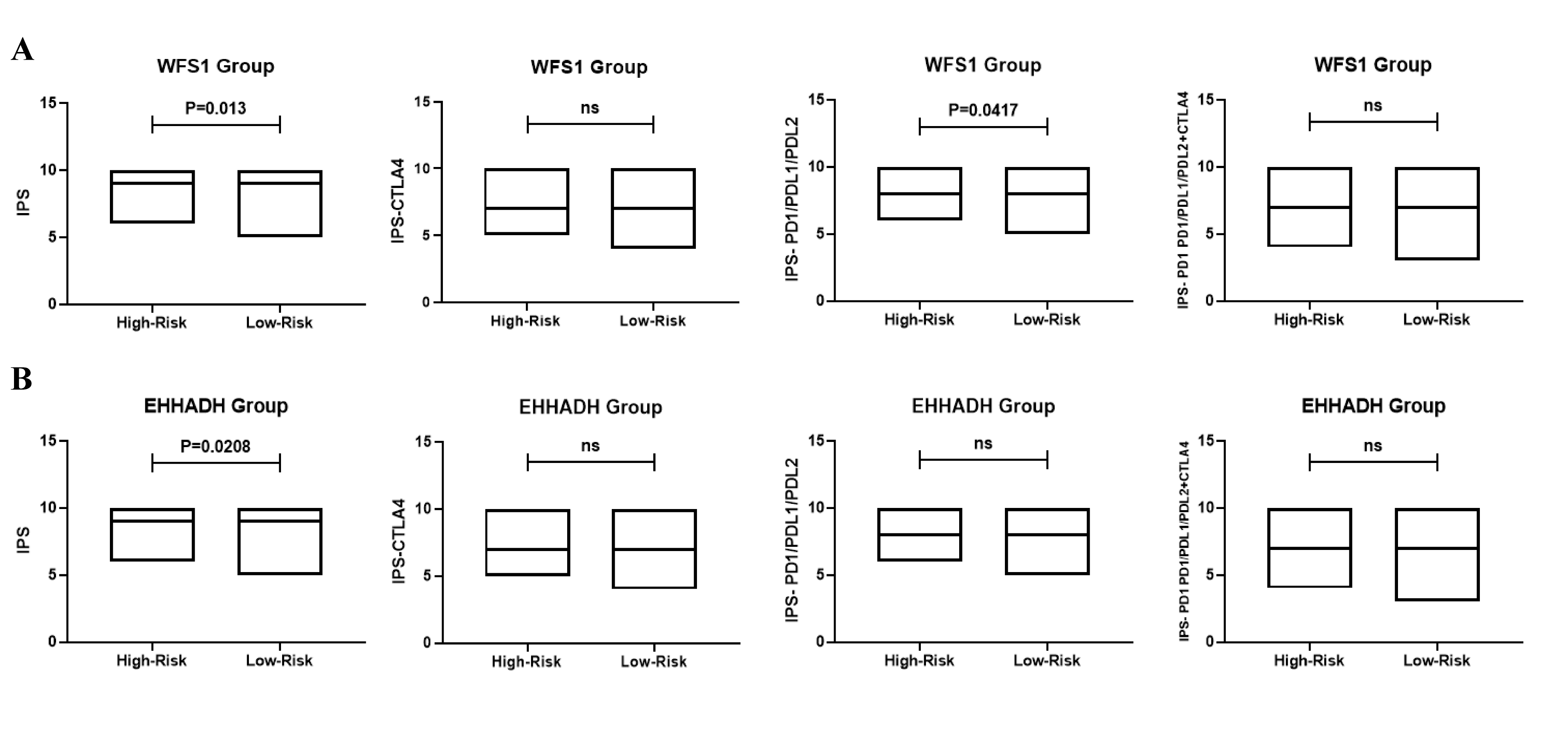

Supplement: Supplementary Figure 1 — Comparison of WFS1 and EHHADH response to immunophenoscore treatment. (A) IPS, IPS- CTLA4, IPS- PD1/PDL1/PDL2, and IPS- PD1 PD1/PDL1/PDL2+CTLA4 in WFS1 signature high- risk group and low- risk group. (B) IPS, IPS- CTLA4, IPS- PD1/PDL1/PDL2, and IPS- PD1 PD1/PDL1/PDL2+CTLA4 in EHHADH signature high- risk group and low- risk group. [file Image_1.tif]
