# Supplementary material for: Identification and Validation of an 6-Metabolism-Related Gene Signature and Its Correlation With Immune Checkpoint in Hepatocellular Carcinoma
Source: Front Oncol. 2021 Nov 15;11:783934. doi: 10.3389/fonc.2021.783934 (PMC8634254; doi:10.3389/fonc.2021.783934)
Supplement: Supplementary file 2 [file DataSheet_1.docx]

https://www.jianguoyun.com/p/DSAHpu8Qo_vhCRiDnYkE
