# Supplementary material for: Identification and Validation of an 6-Metabolism-Related Gene Signature and Its Correlation With Immune Checkpoint in Hepatocellular Carcinoma
Source: Front Oncol. 2021 Nov 15;11:783934. doi: 10.3389/fonc.2021.783934 (PMC8634254; doi:10.3389/fonc.2021.783934)
Supplement: Supplementary file 3 [file DataSheet_2.docx]

Tag logs

0 Q#10313576#9699250#10109482#

1 input=/pub1/data/tmp/Rtmp/7dcae7e4e6bbc0308a755b057b05361c,clinical=/pub1/data/tmp/Rtmp/4ed23c3ed54cf533b37b8740ab6063a8,type=cox,cutoff=auto,minColor=0000ff,medColor=ffffff,maxColor=ff0000,width=10,height=10,platform=linux,outfolder=/pub1/data/user_data/13904059487/cox_het_plot,help=FALSE

2 reading:/pub1/data/tmp/Rtmp/7dcae7e4e6bbc0308a755b057b05361c

3 readed,row:6,column:374

4 readed sample,row:372,column:3

5 common sample:372

6 run cox samples0:372

7 run cox samples1:372

8 run create cox

9 run plot cox

10 save plot

11 save data
