# Supplementary material for: Identification and Validation of an 6-Metabolism-Related Gene Signature and Its Correlation With Immune Checkpoint in Hepatocellular Carcinoma
Source: Front Oncol. 2021 Nov 15;11:783934. doi: 10.3389/fonc.2021.783934 (PMC8634254; doi:10.3389/fonc.2021.783934)
Supplement: Supplementary file 4 [file DataSheet_3.docx]

**Supplementary Table 1. Correlation analysis between 6 MRGs expression and immune cells in HCC.**

| **Name**  **Correlation** | **GAD1** | **SPP1** | **WFS1** | **GOT2** | **EHHADH** | **APOA1** |
| --- | --- | --- | --- | --- | --- | --- |
| **NK cell activated** | **r=0.177**  ***P*=9.82E-04** | r =-0.05  *P*=3.50E-01 | **r =0.155**  ***P*=3.91E-03** | r =-0.078  *P*=1.47E-01 | **r =--0.131**  ***P*=1.51E-02** | **r =--0.135**  ***P*=1.24E-02** |
| **NK cell resting** | **r=-0.135**  ***P*=1.20E-02** | r=-0.069  *P*=2.0E-01 | r =0.094  *P*=7.99E-02 | **r =0.141**  ***P*=8.94E-03** | **r =0.173**  ***P*=1.29E-03** | **r =-0.027**  ***P*=6.21E-01** |
| **Tregs** | r =0.074  *P*=1.71E-01 | **r =0.25**  ***P*=2.51E-06** | **r =0.248**  ***P*=3.03E-06** | **r =-0.221**  ***P*=3.54E-05** | **r =-0.327**  ***P*=4.73E-10** | **r =-0.064**  ***P*=2.38E-01** |
| **T cell gamma delta** | r =0.004  *P*=9.38E-01 | r =-0.063  *P*=2.40E-01 | **r =-0.114**  ***P*=3.49E-02** | r =-0.079  *P*=1.42E-01 | r =-0.013  *P*=8.10E-01 | r =0.095  *P*=7.76E-02 |
| **T cell follicular cell** | **r =0.222**  ***P*=3.23E-05** | r =0.027  *P*=6.22E-01 | **r =0.126**  ***P*=1.90E-02** | **r =-0.215**  ***P*=5.86E-05** | **r =-0.276**  ***P*=1.92E-07** | **r =-**0.065  ***P*=2.28E-01** |
